# Recent Development of Gold Nanoparticles as Contrast Agents for Cancer Diagnosis

**DOI:** 10.3390/cancers13081825

**Published:** 2021-04-11

**Authors:** Dong Luo, Xinning Wang, Clemens Burda, James P. Basilion

**Affiliations:** 1 Department of Radiology, Case Western Reserve University, Cleveland, OH 44106, USA; dxl576@case.edu; 2 Department of Biomedical Engineering, Case Western Reserve University, Cleveland, OH 44106, USA; xxw171@case.edu; 3 Department of Chemistry, Case Western Reserve University, Cleveland, OH 44106, USA

**Keywords:** gold nanoparticles, contrast agents, cancer diagnosis, active targeting, theranositics

## Abstract

**Simple Summary:**

The development of nanotechnology has brought revolution to the diagnosis and therapy of diseases, with a high precision and efficacy. Because nanoparticles can integrate multifunctions together including imaging, targeting, and therapeutics, they are more efficient than the standalone diagnostic or therapeutic entities. Among which, gold nanoparticles are most extensively investigated due to their excellent biocompatibility, versatility and ease of functionalization. Excepting the using of gold nanoparticles as vehicles for therapeutics delivery, they are also good candidates as contrast agents for imaging diagnosis, from magnetic resonance imaging, CT and nuclear imaging, fluorescence imaging, photoacoustic imaging to X-ray fluorescence imaging. We summarize their recent applications in these imaging modalities and challenges for their clinical translation.

**Abstract:**

The last decade has witnessed the booming of preclinical studies of gold nanoparticles (AuNPs) in biomedical applications, from therapeutics delivery, imaging diagnostics, to cancer therapies. The synthetic versatility, unique optical and electronic properties, and ease of functionalization make AuNPs an excellent platform for cancer theranostics. This review summarizes the development of AuNPs as contrast agents to image cancers. First, we briefly describe the AuNP synthesis, their physical characteristics, surface functionalization and related biomedical uses. Then we focus on the performances of AuNPs as contrast agents to diagnose cancers, from magnetic resonance imaging, CT and nuclear imaging, fluorescence imaging, photoacoustic imaging to X-ray fluorescence imaging. We compare these imaging modalities and highlight the roles of AuNPs as contrast agents in cancer diagnosis accordingly, and address the challenges for their clinical translation.

## 1. Introduction

Nanotechnology, which offers unique features and possibilities suited for biomedical applications, has undergone rapid development over the last few decades [1,2,3,4,5]. It brings us huge advantages in the treatment of diseases with superior precision and efficacy, and also accelerates the evolution of new approaches for cancer theranostics. Nanoparticles as theranostic agents that can integrate the functions of imaging, targeting, and therapeutics in a single vehicle, are advantageous over the standalone diagnostic or therapeutic entities in the clinic. They are also easy to deliver to cancerous tissues through the characteristic leaky blood vessels and compromised lymphatic system of the tumor via either passive (EPR effect) alone or combined with active targeting strategies. The EPR effect enhances the entry and accumulation of nanoparticles in tumors and thus a prominent efficacy. Recent work by Luo et al. compared nanoparticles and small molecule delivery of photosensitizers and demonstrated that nanoparticles have a much longer retention in the circulation and the tumor. Additionally, PDT activation can promote the nanoparticles accumulation in tumor by increasing the leakage of blood vessels resulting in further uptake of drug containing AuNPs [6].

Gold nanoparticles (AuNPs) have piqued great interest in the diagnosis and therapy of cancers, owing to their intrinsic properties [7,8,9]. AuNPs are very stable and nonimmunogenic in vivo and have a low toxicity [10]. By exploiting passive targeting, commonly known as the enhanced permeability and retention (EPR) effect, or active targeting [11,12], the preferential accumulation of AuNPs in tumors, may lead to higher sensitivity of imaging diagnosis and increased efficacy of therapeutics. Moreover, AuNPs stabilized with PEG or zwitterions display excellent blood circulation in vivo [13], extending the half time and therapeutic window compared to the small molecular counterparts [14,15]. Finally, synthesis and manipulation of the particle’s physicochemical properties is relatively straightforward. 

AuNPs, depending on the particle size and crystal structure, are classified as clusters with diameters of just a few nanometers or colloidal particles with diameters ranging from a few to several hundreds of nanometers [16]. The size of AuNPs is determined by the synthesizing condition and can be tuned by the reaction temperature, the ratio of reactants, pH, and solvent. The classical synthesis method for gold nanoparticles was introduced by Turkevich, with the reaction of chlorauric acid (HAuCl_4_·4H_2_O) and sodium citrate in an aqueous solution [17]. With this method, the particle size can be precisely controlled from 2 nm to over 200 nm with a seeded growth strategy. By focusing on the inhibition of secondary nucleation during the homogeneous growth process, the enlargement of pre-synthesized AuNPs via the surface-catalyzed reduction of Au^3+^ by sodium citrate can be achieved [18]. Another commonly used protocol is based on a Brust–Schiffrin method, which uses a two-phase toluene-H_2_O system [19]. Using this method particle size can be adjusted between 1.5 and 5.2 nm by varying the Au:dodecanethiolate ratio, reaction temperature, and rate at which the reduction is conducted [20]. To synthesize gold nanoclusters, a biomineralization process is utilized by using peptides or proteins as scaffolds to sequester and interact with inorganic ions, and the reduction of ions is achieved by raising the pH [21,22]. The ultrafine nanocluster structure leads to molecular-like properties. Different AuNPs shapes, such as rods [23], shells [24], triangles [25], and stars [26], can also be easily achieved with unique chemical, electrical and optical properties. However, most examples of diagnostics using gold nanoparticles seem to utilize AuNPs likely because of their size can be tuned to around 1–2 nm level, which is hardly achievable for nano shells, stars, cages or any other shapes, and can penetrate tumors more deeply. Further, AuNP ultrasmall size is key for the clinical translations to avoid unnecessary retention in the body and ensuing toxicity. The basic features of AuNPs with different shape and sizes, their synthesis routes, and properties are very well summarized by Tabish et al. [27], so they will not be discussed in detail here. The size and shape of AuNPs have a fundamental influence on their biomedical applications. For instance, gold nanorods are used for photothermal therapy due to their absorbance of the near-infrared light [28,29]. Radiosensitizing ability of AuNPs is also directly dependent on the particle size [30], and AuNP size will also affect the AuNPs intracellular and in vivo distribution [31,32]. Smaller-sized nanoparticles more easily penetrate into tumor tissues but a study by Liang et al. indicated that 12 nm AuNPs led to better tumor inhibition upon radiation compared with either 5 nm, 27.3 nm, or 46.6 nm AuNPs [33]. These studies revealed the impact of AuNPs core size on their application as radiosensitizers, but also demonstrated that different particle surface functionalization also affects efficacy, making direct comparisons of particle efficacy, based on particle size alone, difficult. 

AuNPs surface functionalization is necessary to reduce the surfactant-induced toxicity and increase their biocompatibility for biomedical applications [34,35]. The most commonly used modification is PEGylation, which uses thiol terminated polyethyene glycol (PEG) to exchange the surfactant, taking advantage of the Au-S covalent bonding achieved by the thiolated PEG ligand [36]. It is well known that PEG is beneficial in enhancing the solubility, reducing nonspecific binding, and thus can improve the biocompatibility and circulation half-life of AuNPs [37]. However, there are some drawbacks to PEG as the injection of PEGylated NPs may induce humoral immune responses, and thus accelerate blood clearance for the second injection of NPs [38]. More recently, Tang et al. reported that Au-Se bonds exhibit superior anti-interference properties in the presence of glutathione (GSH) compared with the Au-S bonds, which may offer a new perspective in future AuNPs design to enhance their stability and selectivity for the in vivo applications [39]. The AuNPs can also be stabilized by zwitterionic ligands [40] or other natural biomacromolecules, such as DNA and protein reducing negative effects of other coating such as PEG [41,42]. Depending on the protective coating, the interaction between AuNPs and the biological environment can be adjusted. Luo et al. have demonstrated a DNA functionalized AuNPs that uses a DNA strand as the fuel and Exo III as the energy-dissipating element to manipulate four-dimensional AuNPs assembly by adding an additional dimension: time, which may regulate their interaction with living cells [43]. Active-targeted AuNPs are more likely to penetrate deeply into tumors and have a longer retention compared to the passive targeted AuNPs [44,45]. The ease of AuNPs surface modification by binding thiols and amines provides a convenient way to introduce active targeting ligands on their surface [46,47]. Functionalizing AuNPs with targeting moieties can effectively avoid off-target accumulation and improve the precision of therapeutic delivery. Antibodies and targeting ligands, such as folic acid, epidermal growth factor receptor I (EGFR), and RGD, have been conjugated to AuNPs for targeted tumor imaging or chemotherapy drug delivery [48,49,50,51]. More recently, Luo et al. have developed a prostate-specific membrane antigen (PSMA) targeted AuNP utilizing a small peptide ligand (PSMA-1), which has a high binding affinity [14,52]. 

The functionalization of AuNPs with active targeting moieties facilitates precise tumor accumulation, and thus potentially improve tumor diagnosis and therapy efficacy. Chemotherapy drugs, such as platinum (IV) and doxorubicin, have been loaded to AuNPs for precise tumor therapy [53,54]. Taking advantage of the unique optical and electronic properties of Au, AuNPs are also exploited for externally triggered release of the pre-loaded therapeutics [55]. Basilion and Burda reported extensive work using AuNPs as carriers to deliver photosensitizers for the photodynamic therapy of brain tumor and prostate cancer [6,50,56]. AuNPs can be also be used for the delivery of other “less conventional” therapeutics, such as genes and small-interfering RNAs for gene therapy [57]. The uses of AuNPs for photothermal therapy, radiotherapy, and as carriers for delivery of cancer therapeutics, are reviewed in detail previously [16,58,59,60,61]. Thus, in this review, we will focus specifically on pre-clinical development for the application of AuNPs in imaging techniques, such as magnetic resonance imaging (MRI), PET/CT imaging, photoacoustic imaging, fluorescence imaging and X-ray fluorescence imaging for cancer diagnosis (Table 1).

## 2. Gold Nanoparticles in Cancer Diagnosis

### 2.1. Magnetic Resonance Imaging (MRI)

Magnetic resonance imaging (MRI) is a noninvasive imaging modality that is frequently used in the clinic for disease diagnosis, molecular imaging, and cell tracking, owing to its high spatiotemporal resolution and excellent soft tissue contrast [72,73,74]. The basic principle of MRI is based on nuclear magnetic resonance (NMR) and the relaxation of proton spins which align either parallel or antiparallel to the applied magnetic field. When a “resonance” frequency in the radio-frequency (RF) range is introduced to the nuclei, the protons are excited to the antiparallel state, and then relax to their initial, lower-energy state after the disappearance of the RF pulse. There are two different relaxation pathways: longitudinal or *T_1_* relaxation, involving the decreased net magnetization (Mz) recovering to the initial state, and transverse or *T_2_* relaxation, involving the induced magnetization on the perpendicular plane (Mxy) disappearing by the dephasing of the spins [75]. Though it is noninvasive and no radiolabels or ionizing radiation are needed, MRI suffers from lower sensitivity compared to other imaging modalities such as fluorescence and bioluminescence imaging and positron emission tomography [61]. Therefore, MR contrast agents are commonly used to enhance the MR contrast sensitivity. Among which superparamagnetic contrast agents typically using superparamagnetic iron oxide nanoparticles (SPIO) have been extensively investigated [76]. However, SPIOs have limited in vivo stability and can generate reactive oxygen species, which may potentially lead to severe DNA and protein damage as well as inflammation [77]. Gold nanoclusters, with diameters of less than 2.5 nm, are considered to be semiconducting quasimolecules and have magnetic properties depending on their protective ligands [78]. Stevens and co-workers have incorporated gold quantum dots into silica particles, preserving their near-infrared photonics and paramagnetism, and used the hybrid particle for MR imaging of colorectal carcinoma [79].

**Figure 1:**
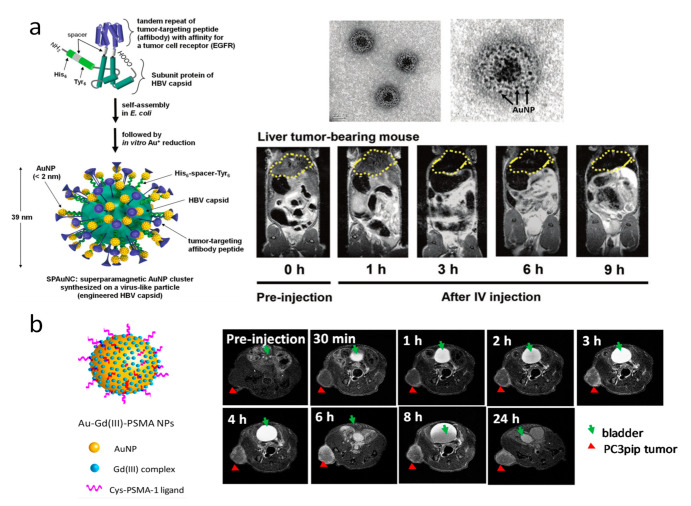
(**a**) Schematics of a SPAuNC and its synthetic procedure (left) and transmission electron microscopy (TEM) analysis of SPAuNCs (top right); *T_2_*-weighted MR images of liver tumor (MDA-MB-468) bearing mice showing the time course of after SPAuNCs injection (down right, liver as indicated by yellow dots). Adapted with permission from Wiley [62]. (**b**) Schematic representation of Au−Gd(III)- prostate-specific membrane antigen (PSMA) nanoparticles (NPs) and time course in vivo *T_1_*-weighted spin echo images of mice showing active targeting of NPs to PC3pip tumor (red triangles). Adapted with permission from American Chemical Society [64].

Recently, Kwon et al. have synthesized superparamagnetic Au nanoclusters (SPAuNCs) with surface distributed AuNCs less than 3 nm, using a tumor targeting virus-like particle as a synthetic scaffold (Figure 1a) [62]. They modified hepatitis B virus (HBV) core protein with NH2-His6-spacer peptide-Tyr6-COOH at the N-terminus, and replaced Pro79Ala80 with the tandem repeat of affibody peptide, which has specific and strong affinity for human epidermal growth factor receptor I (EGFR) [63,80]. The engineered HBV capsids have a diameter of 36.6 nm, and the abundance of His6 and Tyr6 located on the surface was utilized to react with chloro(trimethyl phosphine) gold(I) [81]. A raspberry-like cluster of AuNPs, each 3 nm in diameter, on the engineered HBV capsid were developed. The SPAuNCs showed a good MR contrast for *T_2_*-weighted imaging with *r_2_* to *r_1_* relaxivity ratio (*r_2_*/*r_1_*) of 6.8. The mechanism for the magnetism of the SPAuNCs is due to the chemically bond of small AuNPs to phosphine phosphorus and the near side-chain oxygen of Tyr6. Because of the interaction, the charge transfers from the surface Au atoms of AuNPs to both the phosphine phosphorus and tyrosine oxygen, resulting in the occurrence of unoccupied d states, the interaction between localized and delocalized spins in d-band of the surface Au atoms, and thus the superparamagnetic features of the SPAuNCs [62]. The performance of SPAuNC as a *T_2_*-weighted MR contrast agent was demonstrated with mice bearing liver tumor (MDA-MB-468), showing that the tumorous tissues located in the liver was clearly differentiated from the normal healthy liver tissue. Moreover, due to the small size, the SPAuNCs showed excellent biocompatibility and clearance with less than 1% of the initial Au dose remaining in liver after 42 days compared to 19% for synthetic AuNPs, which is attractive for clinical uses for cancer diagnosis.

The other category of MR contrast agents are paramagnetic, with gadolinium(III) [Gd(III)]-based agents being best known. Gd(III) is usually chelated and can reduce the longitudinal relaxation time of nearby H_2_O protons. Gd(III) chelates have been conjugated to AuNPs, with a surprising relaxivity improvement, and intracellular and intra-tumor retention [82,83]. Studies by Meade and co-workers have conjugated 1,2 diothiolate modified chelates of Gd(III) to AuNPs surface and improved the relaxivity (*r_1_*) up to 4.5-fold compared to the molecular dithiolane-Gd(III) complex [84,85]. The dramatic *r_1_* relaxivity increase is because of the increased local Gd(III) concentration per particle [86]. When they injected (ip) the dithiolane-Gd(III) AuNPs into mice at a Gd(III) dose as low as 8.8 μmol/kg body weight, significant MR contrast enhancement in the pancreas was observed [87]. Meade’s group also found that AuNPs shape had a profound impact on the *r_1_* relaxivity, with greater contrast enhancement for gold nanostars than spheres because of an increased contribution of second-sphere relaxivity [88]. When the dithiolane-Gd(III) AuNPs were conjugated with prostate-specific membrane antigen (PSMA) targeting ligands, there was a remarkable selective uptake of AuNPs by PSMA-expressing prostate cancer cells with excellent MR contrast in vitro and in vivo (Figure 1b) [64]. The targeted AuNPs also showed superior radiosensitizing characteristics amplifying the effect of radiotherapy and show a promising future application for MR-guided radiotherapy [64].

### 2.2. CT and Nuclear Imaging

**Figure 2:**
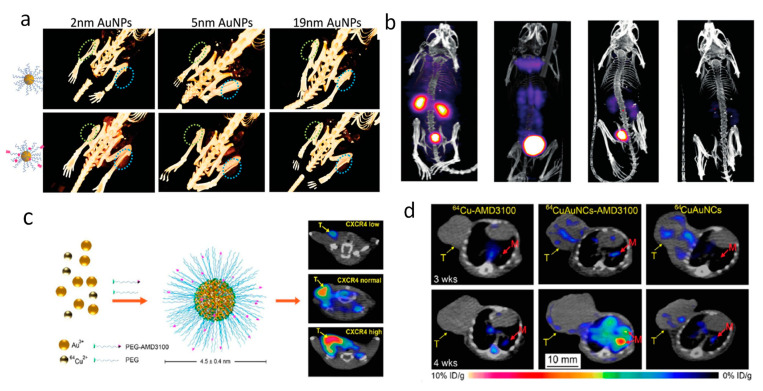
(**a**) Selective tumor accumulation of PSMA-targeted AuNPs (bottom panel) to PSMA-expression PC3pip tumors (blue circles) over non-expressing PC3flu tumors (green circles) with NPs sizes of 2 nm, 5nm and 19 nm *vs.* non-targeted AuNPs with the same sizes (top panel) at 4 h post-injection. Adapted with permission from [65], published by The Royal Society of Chemistry. (**b**) SPECT images of balb/c mice at 10 min, 1 h, 4 h, and 24 h post-injection of GS-[^198^Au]AuNPs. Adapted with permission from [66], published by Wiley. (**c**) Schematic of ^64^Cu-doped AuNCs with AMD3100 targeting (^64^CuAuNCs-AMD3100) for PET imaging of breast cancer. (**d**) PET/CT imaging of breast cancer lung metastasis with ^64^CuAuNCs-AMD3100, ^64^CuAuNCs alone and free ^64^Cu-AMD3100 at 3 and 4 weeks after tumor implantation. Adapted with permission from [68], published by American Chemical Society.

Computed tomography (CT) is a widely used imaging modality in clinic that uses X-rays and a detector array to create cross-sectional images of the body with high spatial and temporal resolution [48,89]. As a noninvasive clinical imaging tool, CT is able to visualize the internal structures of the body, providing three-dimensional anatomical details for diseases diagnosis and therapy prediction. The main drawback for CT imaging is the lack of sensitivity of soft tissues compared to nuclear imaging and MRI. CT contrast agents are used to improve the sensitivity of CT imaging, among which iodine-containing probes and AuNPs are intensively investigated [90]. The mechanism for CT contrast agents is much simpler than MRI. The contrast agents have a higher electron-density than the tissues and thus higher absorbance of X-ray, producing direct contrast effects on their positions. Compared to iodine based contrast agents, which have a fast renal clearance and renal toxicity, AuNPs exhibit 2.7 times larger X-ray mass attenuation than the iodine, making them attractive for CT imaging enhancement [91]. Numerous studies have investigated the possibility of using AuNPs as CT contrast agents to diagnose cancer, taking advantage of the passive targeting ability of NPs via the enhance permeability and retention (EPR) effect, exploiting the relationship of AuNPs size and CT contrast and their in vivo kinetics [90,92]. Active targeting ligands for multiple tumor biomarkers have also been functionalized on the surface of AuNPs to further improve their accumulation and tumor targeting specificity. Blum and co-workers grafted a cathepsin inhibitor GB111-NH_2_ to AuNPs surface and developed an activity-based probe (ABP) for CT imaging of cysteine cathepsin activities in cancerous tissues [89]. Luo et al. reported a prostate-specific membrane antigen (PSMA) targeted AuNPs that are selective to PSMA-expressing prostate cancer cells (PC3pip) and using micro-CT found that the accumulation of PSMA-targeted AuNPs in prostate tumors in mice revealed a size-dependent pattern (Figure 2a) [65]. Other tumor targeting ligands, such as anti-Her2 antibodies (Herceptin) [48], folic acid [93,94] and RGD [51] have also been conjugated to AuNPs to construct active tumor targeting CT contrast agents. Though AuNPs displayed good X-ray attenuation, the long retention time in the body limits their practical applications as a CT contrast agent. Recent efforts are concentrated on developing ultrasmall gold nanoclusters, which display tumor contrast and good renal clearance [95,96]. Recent work by Basilion and co-workers has shown that PSMA-targeted gold nanoclusters have a high tumor selectivity, excellent renal clearance with 14% ID/g Au excreted in urine at 24 h post-injection, and two times less retention in liver compared to the AuNPs counterpart as revealed by ICP and CT imaging [97].

Nuclear imaging techniques, such as single photon emission computed tomography (SPECT) and positron emission tomography (PET), have a much higher sensitivity and accuracy compared to CT imaging. They are very frequently used for diagnosis, especially for cancer. Different from CT imaging, which uses an external X-ray and detector to record the attenuation of signals, nuclear imaging uses radioactive substances to emit radiation and external detectors to capture the radiation emission.

Application of AuNPs in nuclear imaging is also very attractive [5]. Zheng and co-workers were the first who doped ^198^Au into the GSH protected gold nanoclusters via a one-step thermal reduction of ^197^Au and ^198^Au ions mixtures with GSH. The [^198^Au]AuNCs had a hydrodynamic diameter of 3 nm and exhibited a rapid *t_1/2α_* of 5.0 min and *t_1/2β_* of 12.7 h, which was similar to clinical available small molecule contrast agents (Figure 2b) [66]. The [^198^Au]AuNCs had a short half time and fast renal clearance as revealed by SPECT. ^64^Cu is another radiotracer that has been incorporated into AuNPs. Liu and co-workers successfully synthesized ^64^Cu alloyed gold nanoclusters (^64^CuAuNCs) for PET imaging of prostate cancer, with good stability, high renal clearance, and passive tumor targeting [67]. When the ^64^CuAuNCs were functionalized with active targeting ligands (AMD3100), they were able to selectively detect primary breast cancer and the lung metastasis by PET imaging (Figure 2c,d) [68]. Compared to the conventionally used ^64^Cu, which is usually stabilized through chelating with macrocyclic chelators, ^64^Cu doped AuNPs have a more efficient systemic clearance, thus reducing the potential toxicity and unwanted radiation burden [98].

### 2.3. Fluorescence Imaging

**Figure 3:**
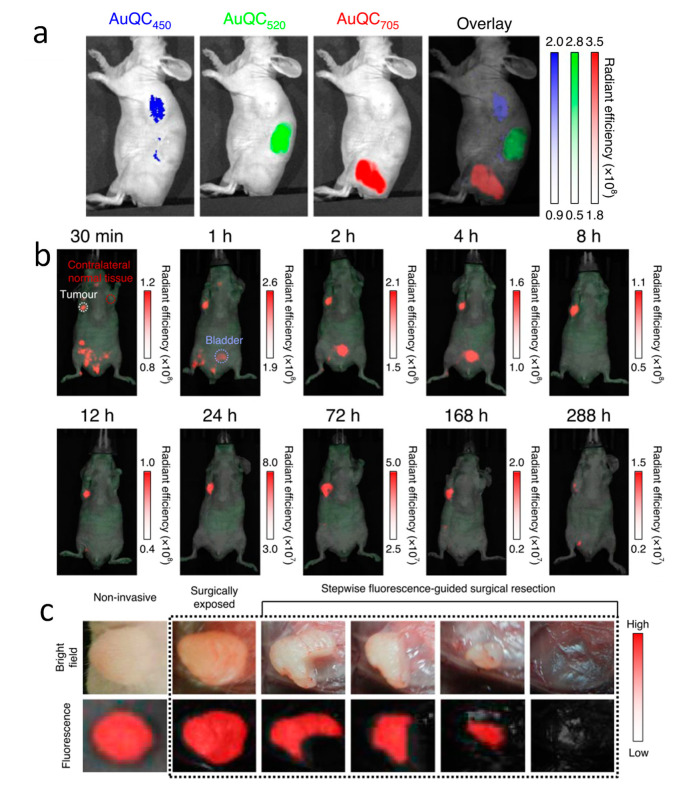
(**a**) In vivo fluorescence imaging of mice after subcutaneous injection of AuNCs with emission at 450 nm, 520 nm, and 705 nm. (**b**) Time course in vivo fluorescence imaging of human breast cancer bearing mice (MDA-MB-231) after injection of AuNC_705_. The renal clearance of AuNC_705_ (blue circle, bladder) and the long retention time in tumor (white circle) are highlighted. (**c**) NIR-fluorescence-guided intraoperative surgery after AuNC_705_ injection, showing the tumor that was surgically exposed and sequentially removed until the entire tumor was completely removed. Adapted with permission from [69], published by Springer Nature.

AuNPs have a unique optical properties, known as surface plasmon resonance, but when the size reduces to the subnanometer scale, gold nanoclusters (AuNCs) start to exhibit photoluminescence [99]. Small nanoclusters are believed to have discrete energy levels, but the exact picture of the discrete energy levels and the nature of quantum effects in AuNCs remained elusive. It is believed that the visible and near-infrared (NIR) fluorescence of AuNCs with a core-shell structure originates from the metal core state and from the surface states of the –SR-Au-SR-Au-SR- staples, respectively [100]. Therefore, AuNCs show great promise as optical imaging agents. The fluorescence of AuNCs is determined by the intrinsic quantization effects of the core and the surface ligands [101]. Sometimes, the synthesis routes will also affect the fluorescence of AuNCs. Yang et al. have synthesized alpha-lactalbumin stabilized AuNCs with three different emission wavelengths of 450 nm, 520 nm, and 705 nm (Figure 3a), and demonstrated good in vivo fluorescence imaging of MDA-MB-231 human breast cancer using the 705 nm AuNCs (Figure 3b), and a possibility for utilizing the AuNCs for fluorescence-guided surgical resection (Figure 3c) [69]. The fluorescence of AuNCs is more stable than molecular dyes and the AuNCs are superior to the molecular dyes as a fluorescence imaging agent. Liu et al. performed a study to directly compare the GSH protected AuNCs and the small molecular dye IRDye 800CW for MCF-7 tumor imaging, and found that GSH-AuNCs had a longer tumor retention time than the IRDye 800CW [102]. In addition, the clearance of GSH-AuNCs from normal tissue was also more than 3 times faster than that of the IRDye 800CW, indicating a superior signal to noise ratio for fluorescence imaging. Compared to the passive targeted of AuNCs mentioned above, Pyo et al. conjugated folate ligands to the AuNCs, enabling selective binding to folate-receptor positive cancer cells, and found that the folate groups further boosted the fluorescence quantum yield of AuNCs by energy transfer [103]. Most of the luminescent AuNCs reported so far have an emission wavelength less than 950 nm, but by tuning the type and stacking structure of the surface ligands, the emission wavelength of AuNCs can be extended up to 1300 nm. Bawendi and co-workers have successfully demonstrated in vivo fluorescence imaging using these long emission-wavelength AuNCs and exhibited improved contrast and resolution due to reduced photon scattering in tissues when comparing to that of conventional NIR (<900 nm) imaging [104]. These studies confirmed the great potential of AuNCs as fluorescence imaging agents for cancer diagnosis. Moreover, due to their optical properties, the AuNCs can be activated by light to produce reactive oxygen species, making them good candidates as photosensitizers for photodynamic therapy of cancers [105,106].

### 2.4. Photoacoustic Imaging

**Figure 4:**
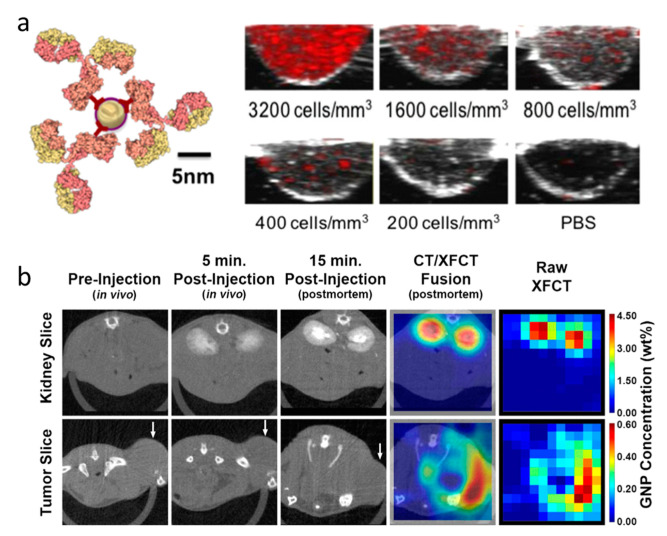
(**a**) Schematic of the epidermal growth factor receptor I (EGFR)-targeted AuNPs and representative Photoacoustic imaging (PAI) cross-sectional images of AuNPs labelled A431 cells at different concentrations. Adapted with permission from [70], copyright 2019 Optical Society of America. (**b**) Time course reconstructed axial CT images after AuNPs injection and X-ray fluorescence computed tomography (XFCT) images at the levels of the kidneys and tumor (white arrow). Adapted with permission from [71], published by Springer Nature.

Photoacoustic imaging (PAI) is a noninvasive imaging modality, which can monitor in real time the anatomical, functional and molecular signals of diseased tissues with a high spatial resolution [107,108]. In PAI, a non-ionizing laser pulse, the energy of which is absorbed by the endogenous chromophores or exogenous contrast agents, causes a rapid thermoelastic expansion of tissue, and thus leading to the generation of a wide-band ultrasound wave [109]. The ultrasound wave can be detected with a transducer and signals will be reconstructed into 2D or 3D optical absorption distribution images. PAI is safer than nuclear imaging because of the absence of ionizing radiation, and compared to fluorescence imaging, PAI has a higher resolution and deeper imaging depth. AuNPs have a strong and tunable optical absorption owing to the surface plasmon resonance (SPR) effect, which occurs when free charges on the surface of AuNPs oscillate with the electromagnetic field, leading to an optical absorption. The resonant frequency can change, depending on the size and shape of the AuNPs. Moreover, AuNPs have a high absorption cross-section, so they can serve as excellent PAI contrast agents [70,110]. These characteristics combined with AuNPs accumulation in tumors via an EPR effect and long retention time have enabled AuNPs to be used for PA imaging of various types of tumors, such as breast cancer, melanomas, and brain tumors [109]. When functionalized with targeting ligands, the specificity of AuNPs can be improved, and thus a high selectivity and sensitivity of PAI can be achieved. Sokolov and co-workers have developed an EGFR-targeted AuNPs for active targeting PAI, and demonstrated that 5 nm AuNPs could work as well as the 40 nm AuNPs with a comparable PA signal (Figure 4a), but 5 nm AuNPs showed outstanding tumor penetration and clearance [70]. Furthermore, the targeted AuNPs also displayed an ability to label circulating cancer cells and enabled PAI monitoring of cancer cell mobility [111]. As a PAI contrast agent, the AuNPs can not only localize cancerous tissue, but also aid cancer therapies by providing sequential monitoring of tumor functional properties before, during and after therapeutic procedures.

### 2.5. X-ray Fluorescence Imaging

X-ray fluorescence (XRF) refers to the XRF photons which are normally excited by a source of monochromatic X-rays, such as a synchrotron source, and it has a long history of use for biological samples [112]. By detecting and analyzing the XRF and scattered photons, the distribution of elements in tissue can be quantified, giving us information about their distribution [71]. This process is known as X-ray fluorescence computed tomography (XFCT), which is considered as a promising approach to obtain information of the identity, quantity, and spatial distribution of elements within imaging objects [113]. XFCT combines the advantages of both X-ray imaging and fluorescence imaging, and exhibits distinct advantages over attenuation/contrast-based imaging modalities such as CT. The high energy of XRF (30–70 keV) makes it easy to penetrate biological tissues, which enables imaging much deeper organs than optical fluorescence imaging [114]. In addition, the feature of XRF that is stimulated from high-Z elements, and not from background tissues, can also significantly improve it sensitivity as an imaging modality. Manohar et al. and Zhang et al. have successfully established a protocol to quantify AuNPs concentration by measuring XRF using a benchtop XFCT device, and demonstrated quantitative distribution of AuNPs in liver, kidney, and tumor accordingly, which was confirmed by ICP-MS measurement (Figure 4b) [71,115]. This technology gives us direct evidence of AuNPs distribution in organs and tumors without sacrificing the animals for measurement and shows a great potential for imaging NPs in vivo. By using the versatile AuNPs as a contrast agent, XRF can also be utilized as an effective imaging modality to diagnosis cancer and give direct evidences of in vivo kinetics of the AuNPs. 

### 2.6. Other Imaging Modelity 

AuNPs can also be used in Raman scattering for cancer diagnosis. Scattered Raman photons are molecularly specific and able to distinguish pathological tissues and cells. Due to the surface plasmon resonance (SPR) effect, AuNPs can amplify the surface enhanced Raman scattering (SERS) signals, and thus give chemically specific information of cancerous lesions [27]. For instance, microcalcifications are associated with breast cancer and Raman spectroscopy has been shown to chemically identify differences in benign and malignant microcalcifications of the breast cancer. However, this approach was only applied to ex vivo phantoms and not to a living system [116].

## 3. Conclusions and Perspectives

In summary, we briefly reviewed the recent breakthroughs of AuNPs and their applications in biomedical imaging for cancer diagnosis. The unique optical and electrical properties of AuNPs have enabled their applications as contrast agents in magnetic resonance imaging, CT and nuclear imaging, fluorescence imaging, photoacoustic imaging, and X-ray fluorescence imaging. To improve the biocompatibility and specificity of AuNPs for cancer imaging, their surfaces are modified with various coatings and functionalized with active targeting ligands, which can specifically direct bind to tumor biomarkers. These surface modifications are vital to ensure a successful application and reduction of the potential toxicity to the body. The physical properties of AuNPs, such as size and shape, also play an essential role in the practical uses. For instance, particle shape has a significant impact on MR sensitivity and *r_1_* relaxivity of the Gd-AuNPs. Their size also influences the contrast of AuNPs for CT and photoacoustic imaging. Gold is biologically inert and chemically stable; thus, gold nanoparticles display good biocompatibility. However, due to the variable synthesis routes, size, shape, surface charge, surface conjugates, considerable unwanted toxic effects may still be induced to the biosystem. For instance, cationic particles are moderately toxic compared to the anionic particles, and PEGylation can greatly improve their biocompatibility and in vivo circulation. Various cytotoxic effects have been described in a size and shape-dependent manner. It is believed that toxicity of AuNPs is induced by the release of cytotoxic ions/radicals, and translocation across the cell membrane into mitochondria. In addition, the internalization of AuNPs into cells, the modification of cellular signaling pathways, and destruction of cells/cell membrane can be other sources of toxicity [27]. AuNPs size, shape, and surface characters will also strongly influence their in vivo biodistribution, tumor uptake, endocytosis effectiveness, and clearance rates. Though a bigger size is beneficial for long blood circulation, it, at the same time, hinders the fast clearance from the body and may lead to long term safety issues to the organs, such as liver and spleen [89]. Numerous studies using AuNPs for cancer imaging or therapy did not exhibit notable toxicity to the animals [11], but the long term effect of AuNPs in the body is uncertain and few studies have investigated a prolonged time period for toxicity. There is trend in recent years to use gold nanoclusters for biomedical theranostics, taking advantage of their fast clearance and minimum retention in organs [117,118]. However, due to the variety of particle sizes, shapes and surface modifications, current knowledge on the toxicity and bioavailability of AuNPs has major uncertainties, and it is challenging to make direct comparisons and universal biosafety predictions for different cell lines, animal models, doses at acute, subacute, chronic, and subchronic levels. A better understanding of those fundamental properties will help to conquer the barriers faced in AuNP clinical translation. 

While significant progress has been made for the uses of AuNPs for cancer theranostics, there are still some challenges remaining to be addressed. AuNPs are very stable in vivo and their accumulation in organs may cause potential toxicity. Therefore, efforts have be focused on reducing the size of AuNPs with new synthesis methods, to make them rapidly cleared via the renal system. The ultrasmall AuNPs or nanoclusters demonstrate efficient renal clearance and biocompatibility and while also bringing new unique physical properties for potential biomedical uses, such as imaging and photodynamic therapy. This development is encouraging, but deep insights into AuNPs clearance from kidney and their interaction with different cells is still needed. In addition, for the imaging of different cancers, AuNPs have been functionalized with targeting ligands, which greatly improve the specificity, but bring new questions on how these ligands affect in vivo NPs behavior and bio–nano interactions. Active targeting can enhance AuNPs accumulation in tumors and thus high diagnosis and therapy efficacy, but the step by step metabolism of the NPs once internalized by cancer cells still needs further investigation. The understanding of these issues will help AuNPs design and their clinical translation. 

Overall, AuNPs have exhibited exciting potential for diagnosis of cancer and can serve as a great platform for cancer therapy. Development of chemistry and material sciences have also brought new opportunities to optimize AuNPs structures and features to tackle the challenges addressed for their biomedical applications. We believe that AuNPs will deliver a new horizon for imaging and treating cancers, eventually fulfilling currently unmet clinical needs. 

## Figures and Tables

**Table 1:** Gold nanoparticles as contrast agents for cancer imaging.

Particle Type	Size	Surface	Imaging Modality	Tumor Models	References
Superparamagnetic AuNCs (SPAuNCs)	3 nm	EGFR	MRI (*T_2_*)	liver tumor (MDA-MB-468)	[62,63]
AuNPs	5 nm	PSMA	MRI (*T_1_*)	prostate tumor (PC3)	[64]
AuNPs	2, 5, 19 nm	PSMA	CT	prostate tumor (PC3)	[65]
[^198^Au]AuNCs	3 nm	GSH	SPECT	-	[66]
^64^CuAuNCs	4.5 nm	AMD3100	PET	breast tumor (4T1)	[66,67,68]
AuNCs	2–6 nm	alpha-lactalbumin	fluorescence	breast tumor (MDA-MB-231)	[69]
AuNPs	5 nm	EGFR	PAI	A431 cells	[70]
AuNPs	1.9 nm	-	XRF	prostate tumor (PC3)	[71]
